# CD73 acts as a prognostic biomarker and promotes progression and immune escape in pancreatic cancer

**DOI:** 10.1111/jcmm.15500

**Published:** 2020-07-09

**Authors:** Qiangda Chen, Ning Pu, Hanlin Yin, Jicheng Zhang, Guochao Zhao, Wenhui Lou, Wenchuan Wu

**Affiliations:** ^1^ Department of General Surgery Zhongshan Hospital Fudan University Shanghai China

**Keywords:** adenosine, CD73, methylation, pancreatic cancer, prognosis, tumour immune

## Abstract

CD73 is a glycosylphosphatidylinositol (GPI)‐anchored protein that attenuates tumour immunity via cooperating with CD39 to generate immunosuppressive adenosine. Therefore, CD73 blockade has been incorporated into clinical trials for cancers based on preclinical efficacy. However, the biological role and underlying mechanism of CD73 in pancreatic cancer (PC) microenvironment and its prognostic impact have not been comprehensively studied. In this article, we found that the expression of CD73 was up‐regulated in PC tissues and patients with higher CD73 expression had poorer overall survival (OS) and disease‐free survival (DFS) in multiple publicly available databases. Higher CD73 expression was significantly associated with its reduced methylation, and only the hypomethylation of CpG site at cg23172664 was obviously correlated with poorer OS. Then, Metascape analysis and GSEA showed that CD73 may play an important role in PC progression and immune regulations. Notably, CD73 was verified to be negatively correlated with infiltrating levels of CD8^+^ T cells and γδ^+^ T cells in both TCGA and GEO cohorts via the CIBERSORT algorithm. In addition, patients with higher CD73 expression also tended to have higher PD‐L1 expression and tumour mutation load. It seemed that CD73 might be a promising biomarker for the response to the anti‐PD‐1/PD‐L1 treatment in PC. In conclusion, these results reveal that CD73 may function as a promotor in cancer progression and a regulator in immune patterns via CD73‐related pathways. Blockade of CD73 might be a promising therapeutic strategy for PC.

## 1 INTRODUCTION

Pancreatic cancer (PC) is the fourth leading cause of cancer‐related death with a five‐year survival rate of approximately 9%. In 2020, 57 600 people are estimated to be diagnosed with PC and 47 050 patients will be predicted to die from this disease in the USA.^1^ So far, surgical resection remains as the only theoretically curative treatment. However, no more than about 20% of PC patients are eligible for surgery because most patients are asymptomatic until the disease reaches an advanced stage.^2^ Although multiple therapeutic strategies such as immunotherapy^3^, ^4^ and targeted therapies^5^ have been tried to improve the survival of PC patients, the effect is still limited. This may be partly due to the complexity and heterogeneity of PC. Therefore, the exploration of a promising biomarker, which regulates biological dynamics and behaviour of PC, will contribute remarkably to optimize treatment.

CD73, a glycosylphosphatidylinositol (GPI)‐anchored protein encoded by the gene NT5E, reacts on the surface of immune cells, stromal cells and cancer cells.^6^ Functionally, CD73 is involved in ATP metabolism, which cooperates with CD39 to generate anti‐inflammatory adenosine from pro‐inflammatory ATP. Adenosine, via interaction with specific G‐protein‐coupled receptors—A1, A2A, A2B and A3, can enhance the suppressive immune cells including myeloid‐derived suppressor cells (MDSCs) and T regulatory cells (Tregs) and attenuate the protective immune cells such as T cells and NK cells to weaken anti‐tumour immunity.^7^, ^8^ In addition to its enzymatic functions, CD73 also acts as a signal and adhesive molecule to regulate growth, drug resistance, migration and invasion.^9^, ^10^, ^11^, ^12^ In summary, these studies have demonstrated that CD73 could affect the microenvironment of tumour via enzyme and non‐enzyme ways, which seemed that blocking CD73 would be a novel promising therapeutic strategy. CD73 is a classic hypoxia‐inducible factor (HIF) target gene, whose expression is up‐regulated by the hypoxia condition as well as TGF‐β, IFN‐α and Wnt signalling in tumour microenvironment.^13^ Several studies reported that CD73 was up‐regulated in various cancers and higher CD73 level was generally associated with worse clinical outcomes,^14^, ^15^, ^16^, ^17^ but in other studies, increased CD73 expression was adversely considered as a favourable prognostic factor, such as epithelial ovarian carcinoma^18^ and breast cancer (stage I‐III).^19^ These controversial results make it quite difficult to assess its prognostic role in PC patients or even its immune‐related effects in tumour microenvironment especially when studies on CD73 in PC remain largely unexplored.

Here, we comprehensively analysed the expression of CD73 in PC and its prognostic role by using databases such as the Oncomine, GEPIA2 and LOGpc (Long‐term Outcome and Gene Expression Profiling Database of pan‐cancers). CD73 expression levels in PC cell lines were validated by the European Bioinformatics Institute (EMBL‐EBI) and Cancer Cell Line Encyclopaedia (CCLE) databases. Moreover, the relationships among the methylation, expression and prognosis of CD73 were analysed. Subsequently, Metascape analysis and GSEA were conducted to investigate the signalling pathways associated with the regulatory mechanisms and network of CD73 in PC. To assess the influence of CD73 on the tumour microenvironment, we further analysed tumour‐infiltrating immune cells (TIICs) related to CD73 expression via CIBERSORT based on TCGA and GEO cohorts. These findings in this study comprehensively shed light on the prognostic value of CD73 and its underlying mechanism in PC.

## 2 MATERIALS AND METHODS

### 2.1 Data acquisition and preprocessing

The transcriptome expression profiles, DNA methylation data, somatic mutation data and corresponding clinical information of PC were downloaded from the Cancer Genome Atlas (TCGA, https://cancergenome.nih.gov/). The expression data in TCGA were HTseq‐FPKM type, including 178 PC samples and four normal samples. DNA methylation data were obtained from the Infinium HumanMethylation450 BeadChip in TCGA, and the methylation degree was presented as beta value. ‘Masked Somatic Mutation’ data were selected and processed through the VarScan software. Tumour mutation burden (TMB) was calculated from the tumour specific mutation genes. Cases with insufficient TNM stage or follow‐up period <30 days were excluded, and finally 168 cases with detailed clinical information from TCGA database were included in the study. To further validate the results from TCGA, the expression data in data set GSE62165 (platform: GPL13667 Affymetrix Human Genome U219 Array) were retrieved from GEO, which included 118 resected PC tissues and 13 control tissues.

### 2.2 Bioinformatic analysis of targeted gene expression

The mRNA expression of CD73/NT5E in PC cell lines was analysed by CCLE (https://portals.broadinstitute.org/ccle)^20^ and EMBL‐EBI (https://www.ebi.ac.uk/gxa/home)^21^ databases, which are both free and open to get a series of bioinformatics programs for sequential analysis.

GEPIA2, a comprehensive online platform, was used to analyse gene expression profiles from TCGA and the Genotype‐Tissue Expression (GTEx) projects.^22^, ^23^ Comparison of targeted gene expression in PC and normal tissues was performed and visualized in a box plot. The cut‐off value of log_2_FC was set as 1, and *P* value was set to .01.

Oncomine (www.oncomine.org), an online data mining platform,^24^, ^25^ was applied to further compare CD73 expression in PC with that in normal tissues. This analysis was drawn on a series of PC study, including Badea Pancreas, Pei Pancreas, Lacobuzio‐Donahue Pancreas 2 and Segara Pancreas. As criteria, 1.5‐fold change and *P* value = .01 were selected as threshold.

### 2.3 Survival analysis

Survival analysis of CD73 was done in GEPIA2 and LOGpc^26^ (a web server for prognosis analysis of pan‐cancers, http://bioinfo.henu.edu.cn/DatabaseList.jsp). Log‐rank *P* value and HRs (hazard ratio‐95% confidence interval) were analysed for overall survival (OS) and disease‐free survival (DFS) of targeted genes and tumour subtypes via ‘survival’ module of GEPIA2 and LOGpc. The expression threshold was set at 50%, and level above the threshold was considered as high expression.

Joint survival analysis was performed by using Survival R package after methylation level data, gene expression data and corresponding survival time were merged into one matrix via the Hash R package.

### 2.4 Functional enrichment analysis via metascape and GSEA

GeneMANIA (http://www.genemania.org), a useful web interface which can generate a list of genes related to target genes though analysis of functional association,^27^ was performed to construct and visualize a gene‐gene interaction network for CD73/NT5E. Then, all genes in the interaction network, constructed by GeneMANIA, were input in Metascape web service to conduct functional enrichment analysis.^28^

GSEA (gene set enrichment analysis) is usually used to analyse and interpret coordinative pathway changes in high‐throughput transcriptomic experiments. To further validate the influence of CD73 expression on pathway‐level changes of PC, GESA was conducted to investigate whether a priori defined set of genes displayed significantly differential expression between high and low CD73 expression groups in TCGA cohort. The gene was significantly enriched, with a normal *P* value <.01 and a false discovery rate (FDR) <.05.

### 2.5 Analysis of immune cell patterns in microenvironment

CIBERSORT was used to analyse the immune cell fractions of all samples from TCGA and GEO. CIBERSORT, an analytical tool developed by Newman et al,^29^ can quantify the infiltrating immune cell fractions based on normalized gene expression profiles. The standardized processed data set of gene expression was uploaded to the CIBERSOFT website (https://cibersort.stanford.edu/index.php), which ran using 100 aligned default signature matrices. To improve the accuracy of the algorithm, Monte Carlo sampling was used for the deconvolution of each sample to get a CIBERSORT p value, and only samples with a CIBERSORT *P* < .05 were considered eligible for analysis.

### 2.6 Statistical analysis

All statistical analyses were operated via R software (version 3.6.2). The optimal cut‐off value of CD73 to assess prognosis was determined by ROC curve. The correlations between CD73 and clinicopathological characteristics were assessed by Pearson's chi‐square test or Fisher's exact test. Cox regression model was the most commonly used approach for analysing survival time data. The univariate analysis, using the technique of Cox regression, was applied to identify factors that could affect clinical outcome. All the factors significant in univariate analysis were incorporated in the multivariate analysis model to find the independent factors that were significantly associated with poor outcome. Pearson's correlation analysis was performed to assess the correlation between expression and methylation level of CD73. Wilcox test was applied to compare the differences between two groups. The *P* value <.05 was considered as a statistical significance.

## 3 RESULTS

### 3.1 CD73 was Up‐regulated in pancreatic cancer

**FIGURE 1:**
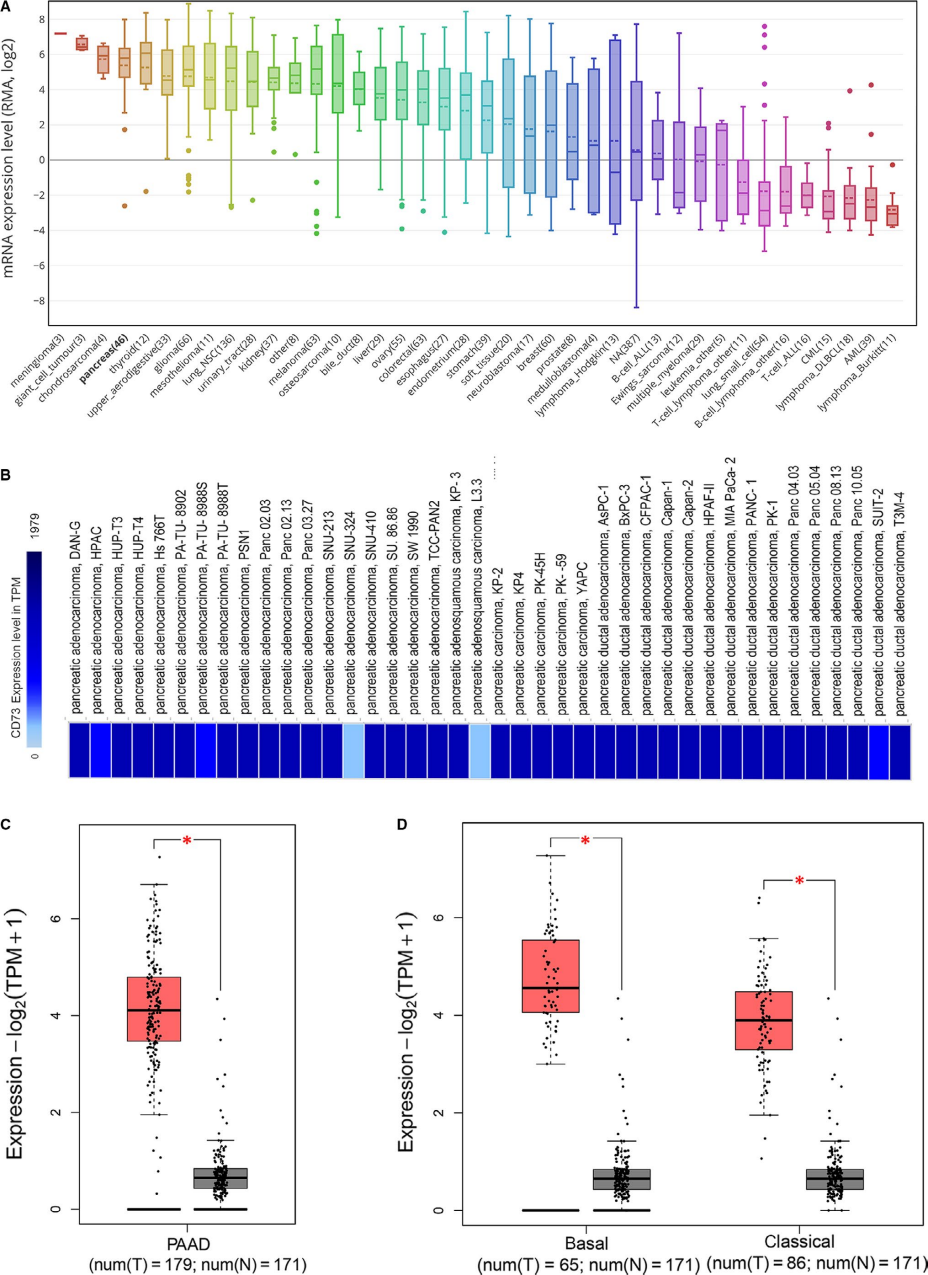
The expression of CD73 in PC cell lines and samples. A, The expression of CD73 in PC cell lines, analysed by CCLE. B, The expression of CD73 in PC cell lines, analysed by EMBL‐EBI. C, Expression of CD73 in PAAD (red) and normal pancreas (grey) based on the TCGA and GTEx data analysed by GEPIA2. D, CD73 expression in PAAD (basal and classical subtypes) and normal tissues based on the TCGA and GTEx data analysed by GEPIA2. *P < .01. CCLE, Cancer Cell Line Encyclopaedia; EMBL‐EBI, European Bioinformatics Institute; TCGA, the Cancer Genome Atlas; GTEx the Genotype‐Tissue Expression; RMA, robust multichip average; TPM, transcripts per kilobase of exon model per million mapped reads; PAAD, pancreatic adenocarcinoma

To determine the expression of CD73 in the PC cell lines and tissues, the following gene expression databases were applied. Analysis of genetic expression data in CCLE revealed that CD73 expression was higher in the PC cell lines than the most of other cancer cell lines (Figure 1A). Moreover, EMBL‐EBI was utilized to validate the expression of CD73 in PC cell lines, which showed that CD73 was high‐expressed in the most PC cell lines (Figure 1B).

The transcriptional levels of CD73 in PC tissues were compared with normal tissues by using GEPIA2. The result revealed that the CD73 expression was elevated in PC tissues (Figure 1C). Considering different gene patterns and prognosis from different heterogeneous subtypes, GEPIA2 was utilized to analyse the expression and prognostic value of CD73 across two PC subtypes (classical and basal subtypes).^23^ We found that CD73 was up‐regulated in the both PC subtypes when compared to normal pancreas tissues (Figure 1D).

Next, the expression of CD73 was further validated with ONCOMINE databases. The findings showed that CD73 mRNA expression was obviously elevated in PC tissues when compared to normal tissues in the Badea Pancreas's data set with a fold change of 3.093 (Figure S1A), the pei pancreas's dataset with a fold change of 2.818 (Figure S1B), the Lacobuzio‐Donahue pancreas 2's data set with a fold change of 3.618 (Figure S1C) and the Segara Pancreas data set with a fold change of 1.751 (Figure S1D).

### 3.2 The prognostic value of CD73 in pancreatic cancer

**FIGURE 2:**
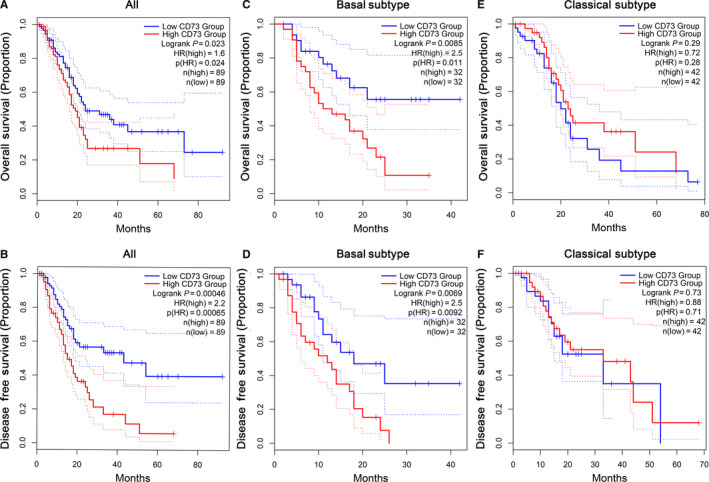
The relationship between CD73 expression and prognosis in PC analysed by GEPIA2. (A) OS and (B) DFS of all PAAD patients in TCGA cohort based on CD73 expression. (C) OS and (D) DFS in PAAD patients with basal subtype based on CD73 expression. (E) OS and (F) DFS in PAAD patients with classical subtype based on CD73 expression. The dotted lines represent the 95% confidence interval. OS, overall survival; DFS, disease‐free survival; PAAD, pancreatic adenocarcinoma

The correlation between CD73 expression and survival in TCGA cohort was next investigated using GEPIA2. Kaplan‐Meier survival analysis disclosed that PC patients with lower CD73 expression had a longer OS than patients with higher CD73 expression (*P* = .023, Figure 2A). Similarly, statistically significant longer DFS was observed for PC patients with lower CD73 expression (*P* < .001, Figure 2B). However, the relationship between survival and CD73 expression was partly different when analysis was stratified into two PC subtypes. A low (<50%) expression of CD73 resulted in statistically significant prolonged OS and DFS for the basal subtypes (*P* = .0085 and 0.0069, respectively, Figure 2C,D). On the contrary, the classical subtype showed no significant correlation between CD73 expression and survival (Figure 2E,F).

To further investigate the relationships between CD73 and other clinicopathological characteristics, patients in the TCGA cohort were divided into the low and high expression groups according to the optimal cut‐off value derived from ROC curve. The correlations between CD73 expression and clinicopathological characteristics of PC were summarized in Table S1. Though there were no clinicopathological features significantly correlated with CD73, it was worth noting that N classification may be strongly associated (*P* = .052) provided that the sample size was enlarged. The univariate analysis showed that tumour site (*P* = .019), T classification (*P* = .029), N classification (*P* = .006), M classification (*P* < .001), adjuvant chemotherapy (*P* = .010) along with CD73 (*P* < .001) were significantly correlated with OS. Furthermore, CD73 expression (*P* < .001) and adjuvant chemotherapy (*P* < .001) remained as independent risk factors for OS in the multivariate analysis (Table 1).

**TABLE 1:** Univariate and multivariate analysis for overall survival in pancreatic cancer patients

Variables	No. (n = 168)	Univariate *P* value	Multivariate *P* value	HR (95% CI)
Age				
≤65	88	1		
>65	80	.224		
Gender				
Female	77	1		
Male	91	.405		
Tumour site				
Head	122	1	1	Reference
Body + tail	27	**.019**	.051	0.480 (0.230‐1.002)
Unknown	19	.912	.444	0.782 (0.417‐1.467)
Grade				
G1	26	1		
G2	91	.675		
G3 + G4	49	.920		
Gx	2	.711		
CD73				
Low	136	1	1	Reference
High	32	**<.001**	**<.001**	2.977 (1.831‐4.839)
T classification				
T1 + T2	28	1	1	Reference
T3 + T4	140	**.029**	.171	1.683 (0.798‐3.547)
N classification				
N0	50	1	1	Reference
N1	118	**.006**	.099	1.644 (0.911‐2.969)
M classification				
M0	164	1	1	Reference
M1	4	**<.001**	.311	2.181 (0.482‐9.866)
Family history				
No	43	1		
Yes	61	.722		
Unknown	64	.767		
Chronic pancreatitis				
No	120	1		
Yes	13	.696		
Unknown	35	.515		
Diabetes history				
No	103	1		
Yes	35	.986		
Unknown	30	.936		
Alcohol history				
No	61	1		
Yes	95	.673		
Unknown	12	.966		
Neoadjuvant therapy				
No	167	1		
Yes	1	.441		
Adjuvant chemotherapy				
No	53	1	1	Reference
Yes	115	**.010**	<.001	0.387 (0.245‐0.610)
Adjuvant radiotherapy				
No	97	1		
Yes	36	.066		
Unknown	35	.080		

The values in bold indicate that they are statistically significant.

Moreover, the prognostic value was further validated by Kaplan‐Meier survival analysis via LOGpc. The results showed that high CD73 expression group had poor OS in the ICGC cohort (*P* < .001, HR = 1.753, Figure S2A), EMTAB6134 cohort (*P* < .001, HR = 1.7759, Figure S2B), GSE62452 cohort (*P* = .0066, HR = 2.2265, Figure S2C) and GSE28735 (*P* = .0188, HR = 2.148, Figure S2D). Therefore, it is convincing that CD73 plays a vital role in influencing the survival of PC patients.

### 3.3 Analysis of CD73 methylation profile in pancreatic cancer

**FIGURE 3:**
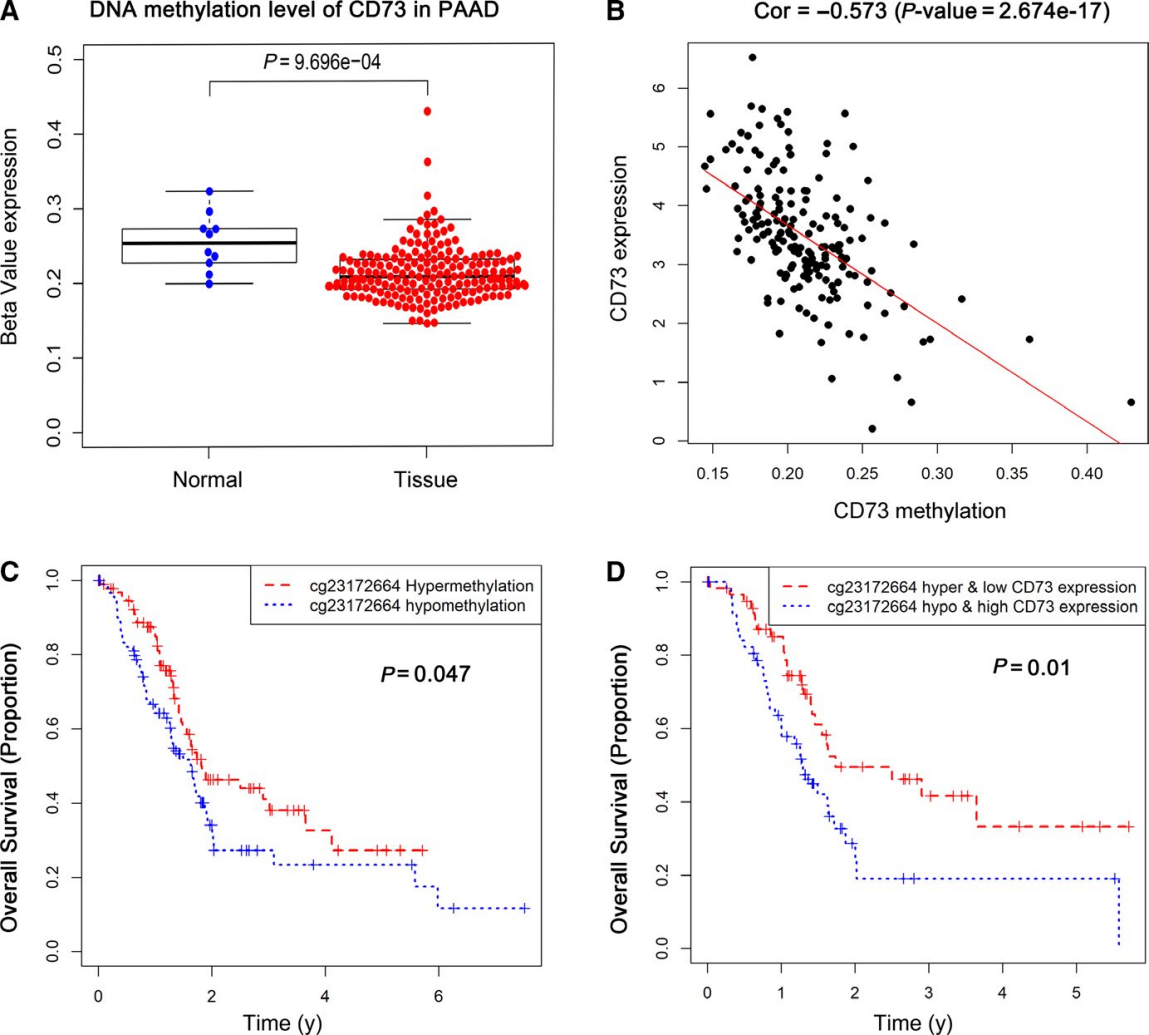
Analysis of the relationships among survival, methylation and expression for CD73. A, The methylation level of CD73 in the normal and PC tissues. B, The correlation between gene expression and methylation of CD73. C, The correlation between CD73 expression and the methylation status of 14 CpG sites. D, The survival analysis of CD73‐related CpG methylation. E, Joint survival analysis of methylation and expression for CD73

To investigate the potential mechanism leading to the aberrant CD73 gene expression, we firstly assessed the level of whole CD73 DNA methylation in 185 PC tissues and 10 normal tissues from TCGA. The methylation level of whole CD73 in PC tissues was lower than normal tissues (*P* < .001, Figure 3A). Then, the correlation between methylation level and expression of CD73 in PC was analysed and showed that CD73 expression was negatively associated with DNA methylation level of CD73 (Cor = −0.573, *P* < .001, Figure 3B).

Furthermore, correlation analysis was performed to identify the association between CD73 expression and methylation of its specific CpG sites (Figure 3C). The methylation level of cg21730993, cg23172664 and cg27039625 showed moderate correlations with CD73 expression (all *r* < −.4, *P* < .001, Figure 3C). Among the above three CpG sites, we found that only patients with the higher methylation level of cg23172664 had a longer OS (*P* = .047, Figure 3D). In addition, combination of cg23172664 methylation degree and CD73 expression showed a better survival stratification for PC patients (*P* = .01, Figure 3E), suggesting that the methylation and expression level of CD73 could influence the prognosis of PC patients.

### 3.4 Interacted genes and enrichment analysis of CD73

A network of CD73/NT5E and its functionally related genes is shown in Figure S3A. Functional enrichment analysis of 21 genes above was conducted via Metascape and the results showed that p38α/β downstream pathway, cellar metal ion homeostasis, pathways in cancer, epithelial cell proliferation, response to inorganic substance were significantly regulated (Figure S3B).

**FIGURE 4:**
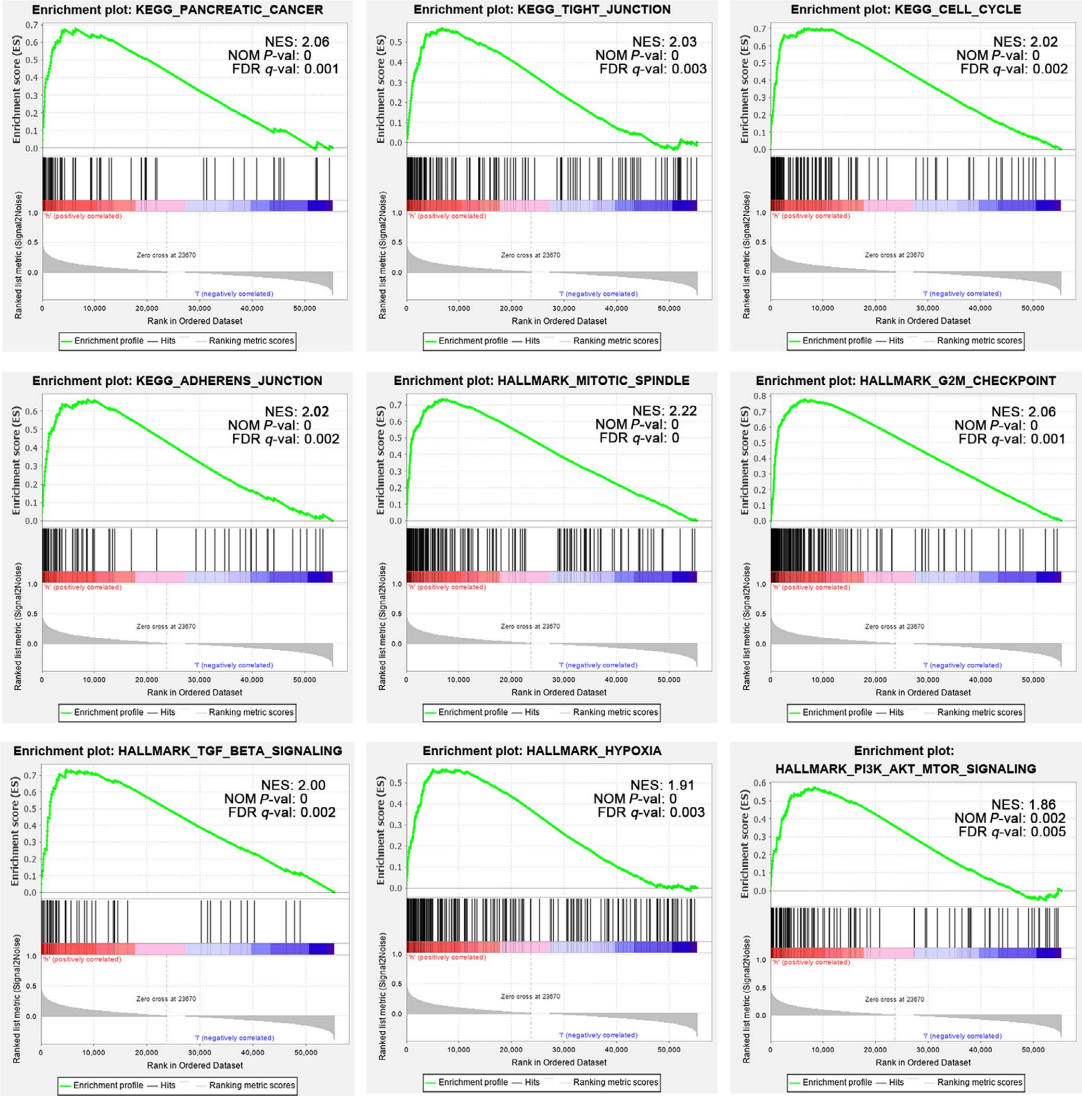
Enrichment plots from GSEA. GSEA results showing pancreatic cancer, cell cycle, tight junction, adherens junction, mitotic spindle, G2M checkpoint, TGF‐β signaling, hypoxia and PI3K‐AKT‐MTOR signalling are differentially enriched in PC cases with high CD73 expression. Gene sets with a normalized *P*‐value <.01 and false discovery rate (FDR) <.05 are considered as significant. GESA, Gene Set Enrichment Analysis; TGF‐β, Transforming growth factor β

To further understand and validate the role of CD73 in PC more definitely, GSEA was conducted between high and low CD73 expression cohorts in TCGA data sets. GESA revealed significant differences (NOM *P*‐val < .01, FDR < 0.01) in enrichment of MSigDB Collection (c2.cp.kegg and h.all.v.7.0.symbols.gmt), and Table S2 showed the details. Gene sets associated to pancreatic cancer, cell cycle, tight junction, adherens junction, mitotic spindle, G2M checkpoint, TGF‐β signalling, hypoxia, PI3K‐AKT‐MTOR signalling were differentially enriched in the CD73 high‐expressed phenotype (Figure 4). So, these findings indicate that CD73 may play an important role in PC progression and immune regulations.

### 3.5 Patterns of tumour‐infiltrating immune cells related to CD73 expression

**FIGURE 5:**
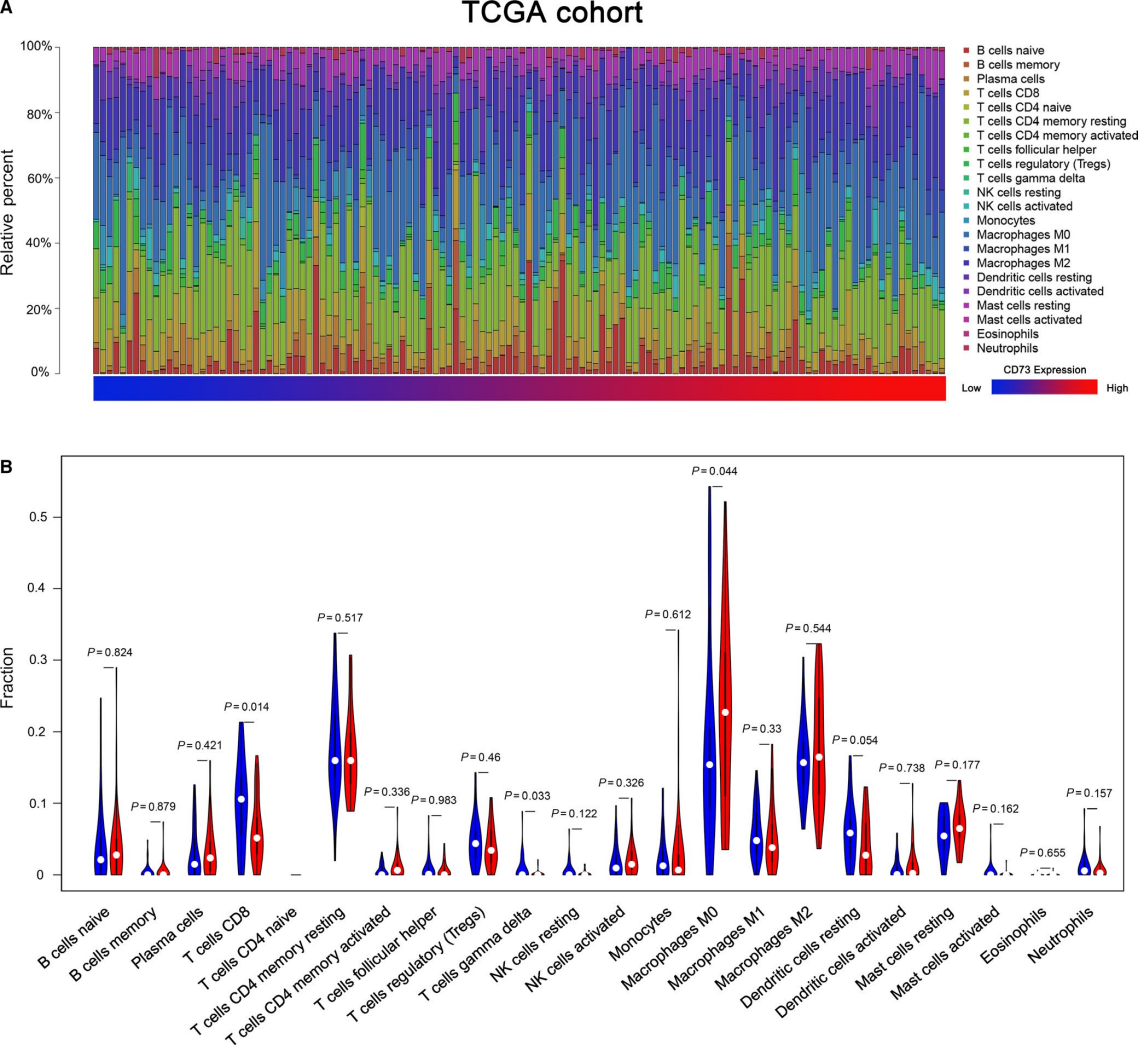
Correlations of CD73 expression with immune infiltration levels in the TCGA cohort. A, The landscape of immune infiltration in 128 tumor tissues arranged by CD73 expression from low to high. B, Analysis of differential immune cells between the low and high CD73 expression group in TCGA

Here, we explored whether CD73 expression was related to immune cells infiltration in PC. After performing CIBERSOFT algorithm, 128 tumour samples in the TCGA cohort with a *P* value < .05 were qualified in this study. The landscape of immune infiltrations in PC obtained from the 128 tumour samples arranged by CD73 expression from low to high was summarized (Figure 5A). To better investigate the effect of CD73 on TIICs, CD73 expression within the last quarter was defined as low expression group, while the top quarter was defined as high expression group. Obviously, the proportions of TIICs varied significantly between both intragroup and intergroup. Compared with low expression group, high expression group contained a lower proportion of CD8^+^ T cells and γδ^+^ T cells, whereas the proportion of M0 macrophages was relatively higher (all *P* < .05, Figure 5B).

To avoid the bias from a limited cohort, GSE62165 was used as a validation data set. 114 tumour samples were eligible after filtering with CIBERSORT *P* < .05. Likely, the landscape of TIICs arranged by the CD73 expression from low to high was summarized in Figure S4A. As same grouping as the TCGA cohort, the proportions of Tregs and activated NK cells were statistically higher in high expression group, whereas infiltrations of CD8^+^ T cells, γδ^+^ T cells and monocytes were higher in low expression group (all < 0.05, Figure S4B). Only CD8^+^ T cells and γδ^+^ T cells were significantly changed in both TCGA and GEO cohort. These results strongly confirm that CD73 has a significant effect in immune infiltration in PC, especially reduction of CD8^+^ T cells and γδ^+^ T cells, which promotes immune escape of PC cells.

### 3.6 Other adenosine‐related genes and correlation with immune checkpoints

**FIGURE 6:**
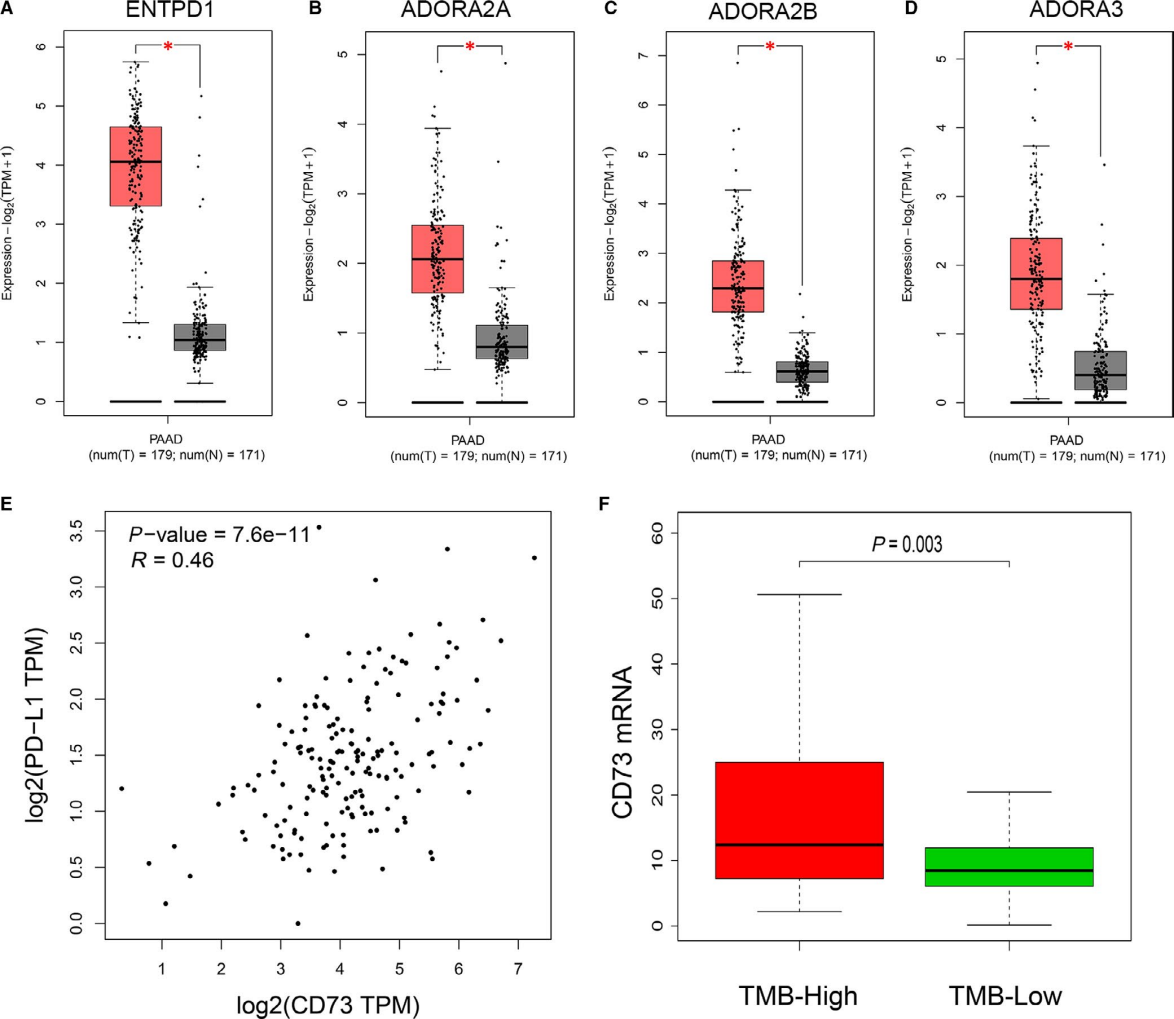
The expression of other major genes on the CD73‐adenosine axis and correlation of CD73 with hot immune checkpoints analysed by GEPIA2. A‐D, Box plots exhibiting the ENTPD1, ADORA2A, ADORA2B and ADORA3 mRNA expression in tumour (red) and normal pancreas (grey) based on TCGA and GTEx data (*P < .01). E, Co‐expression of CD73 and PD‐L1 based on TCGA data. F, Quantitative analysis of CD73 expression in TMB‐High and TMB‐Low groups. TMB, tumour mutation load

ATP undergoes stepwise dephosphorylation by ecto‐nucleotidases including converting ATP to ADP or ADP to AMP by CD39, and dephosphorylating AMP to adenosine by CD73. Thus, CD39 is important for producing adenosine. Gene expression analysis confirmed that ENTPD1 encoding CD39 was also significantly up‐regulated in PC tissues (Figure 6A). Then, the expression of four adenosine receptors (ARs): ADORA1, ADORA2A, ADORA2B and ADORA3 in PC were analysed, and the results indicated that ADORA2A, ADORA2B and ADORA3 were significantly up‐regulated in PC tissues (Figure 6B‐D), which was reported to be associated with tumour immune escape.^8^, ^30^ Thus, over‐activated CD73‐adenosine axis may play an important role in PC progression and immune escape.

Concomitantly, the relationships between CD73 expression and hot immune checkpoints (PD‐L1 and CTLA‐4) were further investigated in PC via GEPIA2. The results showed that a significant positive correlation was observed between CD73 and PD‐L1 expression (*R* = .46, *P* < .001, Figure 6E), but no correlation was observed between CD73 and CTLA‐4 (data not shown). PD‐1/PD‐L1 axis is a recognized co‐inhibitory signalling to promote tumour immune escape in cancers, and the PD‐1/PD‐L1 axis blockage has already received effective clinical responses in several cancers.^31^ Besides PD‐L1 expression, TMB is also a promising biomarker for predicting the response to the anti‐PD‐1/PD‐L1 treatment.^32^ So, TMB was calculated for 150 PC samples and the patients were divided into two groups using the TMB median as the cut‐off value. Surprisingly, the result revealed that CD73 expression was significantly up‐regulated in patients with high TMB in the TCGA cohort (Figure 6F), suggesting high CD73 expression might be a potential predictor for response to the anti‐PD‐1/PD‐L1 treatment.

## 4 DISCUSSION

In this study, we systematically analysed CD73‐related prognosis and TIICs in PC by utilizing online expression databases and bioinformatics data mining tools. In Oncomine and GEPIA2 database, we found that CD73, compared with normal tissues, was highly expressed in PC tissues, and higher CD73 correlated with a poorer prognosis, which was consistent with previous findings in other cancers.^14^, ^15^, ^16^, ^17^ Interestingly, the correlation between CD73 expression and prognosis was partly different when stratified into two PC subtypes, namely classical and basal subtypes. Only PC patients characterized by basal subtype had longer OS and DFS when CD73 was under‐expressed. The basal subtype is more often poorly differentiated and has shorter OS and DFS compared with the classical subtype. The poor survival associated with CD73 expression in this subtype might indicate that CD73 itself and adenosine generated by CD73 are involved in the malignancy of the basal subtype.^33^, ^34^ Next, the univariate and multivariate Cox analyses revealed that CD73 was an independent prognostic factor for OS in the TCGA cohort. What's more, our analysis also indicated that CD73 was mostly high expression in PC cell lines. Several studies have shown that CD73 promotes proliferation, migration, anti‐apoptosis and drug resistance of cancer cells.^35^ Therefore, high‐expressed CD73 may partly result in poor prognosis of PC patients. Meanwhile, we found that CD73 methylation was associated with CD73 expression and OS in PC. Combination of methylation and expression of CD73 had a significant correlation with the prognosis of the PC patients, which has not been reported before to our knowledge and could be worthy of further study.

PC is histologically characterized by the excessive desmoplasia and hypovascularity, which may lead to the hypoxic milieu. Vaupel et al compared the oxygenation of several solid tumours and recognized PC as the most hypoxic.^36^ CD73/NT5E is a classic hypoxia‐inducible factor (HIF) targeted gene, whose expression and function are up‐regulated by hypoxic condition.^37^ Therefore, hypoxia in PC may lead to higher CD73 expression relative to other types of cancer. In addition to hypoxia, TGF‐β can also induce the expression of CD73,^38^ which is consistent with the results of our GSEA analysis. Recently, several studies have shown that the adenosine generated by CD73 can promote metastasis process in some cancers.^35^ A correlation between CD73 expression and metastasis‐related marker was reported.^39^ CD73 suppressed lymphocyte trafficking to metastatic site might be another mechanism to promote metastasis.^40^ However, CD73 expression was not correlated with metastasis in the TCGA cohort, which may be due to the extremely limited cases with metastasis. Expanding the sample size for further study is needed. Meanwhile, CD73 itself may also be engaged in regulating cell cycle to promote the proliferation of cancer cells,^9^ supporting the findings of GSEA and Metascape analysis.

Moreover, CD73 was found to accelerate the adenosine accumulation in the tumour microenvironment, which was considered as a mechanism for cancer immune escape.^35^ To investigate the effect of adenosine generated by CD73 on TIICs in PC, the CIBERSORT was used to compare the immune cell infiltrations between low and high CD73 expression groups. We found that the abundances of immune cells in PC varied significantly between both intragroup and intergroup. Considering the different infiltrations of M0 macrophages and monocytes in TCGA and GEO cohorts, these changes of subtype cells may just exist in independent experiments. Only the immune cells significantly changed in both TCGA cohort or GEO cohort was selected for further discussion to minimize bias and enhanced credibility of the results. Among 22 immune cells, both infiltration of CD8^+^ T cells and γδ^+^ T cells were significantly lower in the high CD73 expression group, which may be involved in the immune evasion induced by CD73 and inhibit tumour immune response.

CD8^+^ T cells are crucial for the protective immunity against tumour. In PC, the infiltration of CD8^+^ T cells in the tumour microenvironment positively correlates with increased OS.^41^, ^42^ Thus, it can be seen that the abundance of CD8^+^ T cells infiltration has significant implication for prognosis and treatment strategies. γδ^+^ T cells, as a bridge between the innate and adaptive immune, are a subgroup of T cells containing T‐cell receptor (TCR) γ and δ chains. It has the ability to produce the cytokines with anti‐tumour effect as IFN‐γ and TNF‐α against cancer cells via stimulating macrophages and DCs and recognize the antigens of tumour cells without major histocompatibility complex restriction.^43^ CD73 may decrease the infiltration of CD8^+^ and γδ^+^ T cells through the following mechanisms: (a) the adenosine generated by CD73 can decrease the function of anti‐tumour T cell and promote T‐cell apoptosis. (b) DCs and macrophages can be polarized into immunosuppressive regulator cells, which limits their stimulation of T cells. (c) A2A receptor activation on Tregs and MDSCs elicits cell expansion and increases their immunosuppressive activity. (d) CD73‐generating adenosine suppresses the adhesion of T lymphocytes to cancer cells. Most of these effects are caused by adenosine binding to its receptors.^8^ Thus, we presented an investigation into the expression of other major genes on the adenosine axis based on patient datasets from TCGA and GTEx project. The expression of ADORA2A, ADORA2B and ADORA3 was significantly up‐regulated in PC tissues. The ARs encoded by the above genes were also validated to be involved in progression and immune evasion of cancers. For example, triggering of ARs, including A1R, A2AR, A2BR and A3R, on the surface of cancer cells could stimulate cell proliferation via activation of AKT, ERK1/2, JNK and P38.^8^ Furthermore, mice devoid of A2AR or administration of A2AR antagonists contributes to inhibiting tumour growth, metastasis, and even complete rejection,^44^ which might also partly due to more infiltration of CD8^+^ and γδ^+^ T cells. So blockade of ARs may be an effective therapeutic strategy for PC as long as confirmed by fundamental and clinical research.

To date, several potent inhibitors and antibodies of CD73 have been discovered and shown the favourable anti‐tumour effects in preclinical studies of other cancers.^45^, ^46^ Recently, some researchers have found that the expression of CD73 in cancer cells limited the efficacy of immune response to immune checkpoint inhibitors and blockade of CD73 enhanced the anti‐tumour activity of anti‐PD‐1/PD‐L1.^45^ The underlying causes deserve further investigation. TMB is commonly defined as the total number of exonic somatic mutations and associated with the number of neoantigens that potentially trigger anti‐tumour immunity. High TMB may be new biomarker predict the responsiveness to immune checkpoint blackade.^32^ Our analysis showed that CD73 expression was positively correlated with PD‐L1 and significantly up‐regulated in patients with high TMB. It seems that CD73 might be a promising biomarker for the response to the anti‐PD‐1/PD‐L1 treatment in PC, and combination of CD73 blockade and anti‐PD‐ L1/PD‐1 mAb might be also a promising therapeutic strategy for PC. Further studies are needed to validate the relationship between CD73 and immune escape. Moreover, several investigators have indicated that targeting CD73 also increased anti‐tumour efficacy of radiotherapy^47^ as well as several chemotherapeutic drugs.^11^ Now several clinical trials combining CD73 blockade with immune checkpoint blockade or classic approaches are recruiting and/or underway.^8^ The results of current trials are worth waiting for, especially in PC.

## 5 CONCLUSIONS

Our findings demonstrate that CD73 can act as a prognostic biomarker and play an essential role in PC progression and immune escape via impairing T‐cell infiltrations and immune response and then results in an unfavourable prognosis, which may lay the foundation for further research of targeting.

## CONFLICT OF INTEREST

The authors confirm that there are no conflicts of interest.

## AUTHOR CONTRIBUTION

**Qiangda Chen:** Conceptualization (equal); Data curation (equal); Formal analysis (lead); Methodology (lead); Validation (equal); Writing‐original draft (equal); Writing‐review & editing (equal). **Ning Pu:** Conceptualization (equal); Data curation (equal); Methodology (supporting); Project administration (equal); Supervision (equal); Validation (equal); Writing‐original draft (equal); Writing‐review & editing (equal). **Hanlin Yin:** Data curation (equal); Resources (equal); Validation (equal); Writing‐original draft (supporting). **Jicheng Zhang:** Data curation (equal); Methodology (supporting); Resources (equal); Validation (equal); Writing‐original draft (supporting). **Guochao Zhao:** Methodology (supporting); Resources (equal); Supervision (equal); Validation (equal); Writing‐original draft (supporting). **Wenhui Lou:** Funding acquisition (equal); Project administration (equal); Supervision (equal); Validation (equal); Writing‐original draft (supporting); Writing‐review & editing (supporting). **Wenchuan Wu:** Conceptualization (equal); Funding acquisition (equal); Project administration (equal); Supervision (equal); Validation (equal); Writing‐original draft (supporting); Writing‐review & editing (equal).

## Supporting information

Figures S1‐S4

Table S1

Table S2

## Data Availability

Publicly available data sets were analysed in this study. The authors confirm that these data can be found here: https://portals.broadinstitute.org/ccle, https://www.ebi.ac.uk/gxa/home, https://www.oncomine.org, http://gepia2.cancer‐pku.cn, http://bioinfo.henu.edu.cn/DatabaseList.jsp, http://www.genemania.org, https://cancergenome.nih.gov and https://www.ncbi.nlm.nih.gov/geo.
