# Supplementary material for: CD73 acts as a prognostic biomarker and promotes progression and immune escape in pancreatic cancer
Source: J Cell Mol Med. 2020 Jul 9;24(15):8674–86. doi: 10.1111/jcmm.15500 (PMC7412695; doi:10.1111/jcmm.15500)
Supplement: Supplementary file 1 — Figures S1‐S4 [file JCMM-24-8674-s001.pdf]

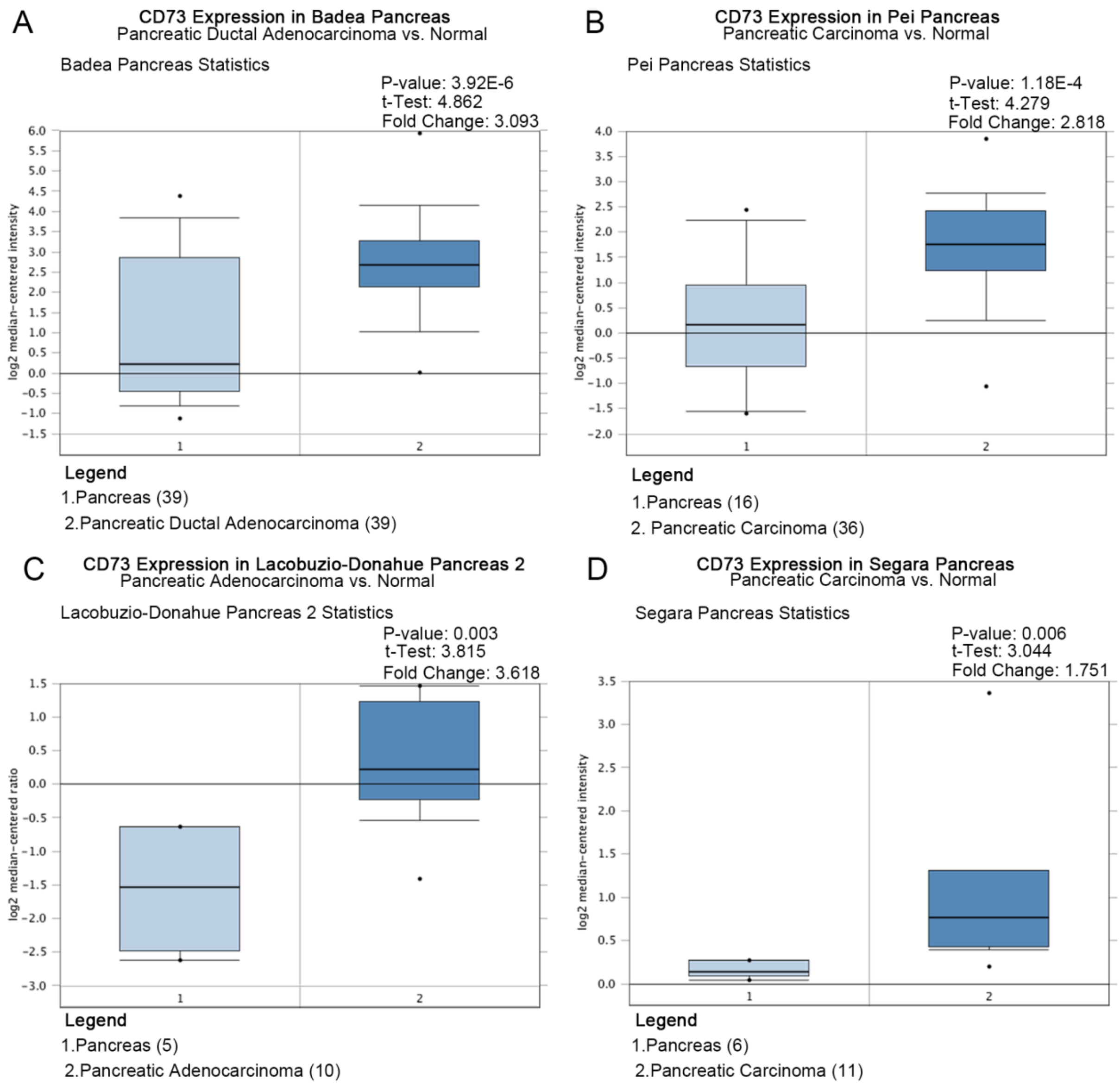

Figure S1. CD73 transcription in pancreatic cancer from Oncomine. Box plot showing CD73 mRNA levels in the (A) Badea Pancreas, (B) Pei Pancreas, (C) Lacobuzio-Donahue Pancreas 2 and (D) Segara Pancreas datasets.

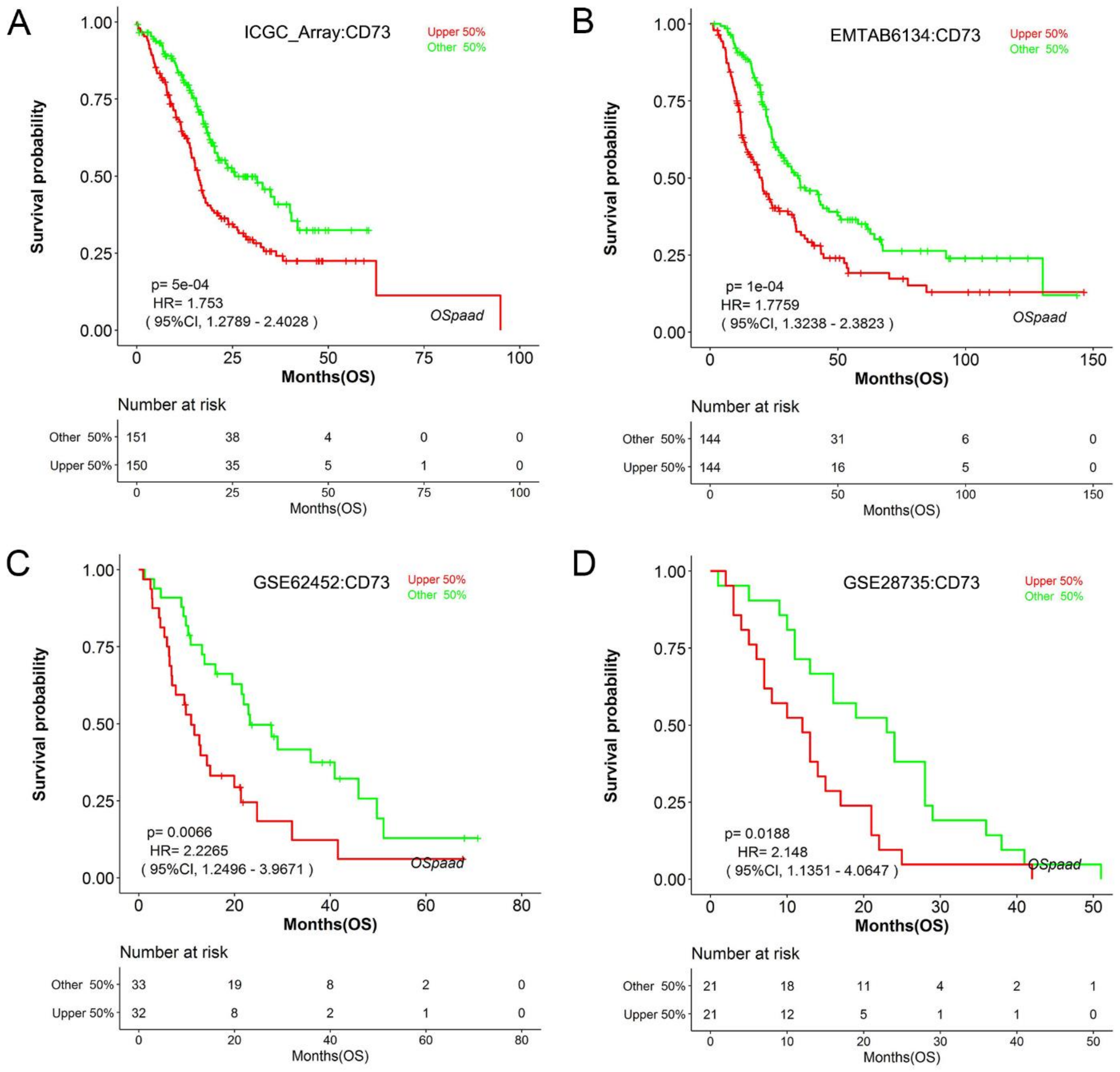

Figure S2. Prognostic value of CD73 in pancreatic cancer analyzed by LOGpc. Kaplan-Meier survival curves of overall survival in the (A) ICGC, (B) EMTAB6134, (C) GSE62452 and (D) GSE28735 datasets based on CD73 mRNA expression.

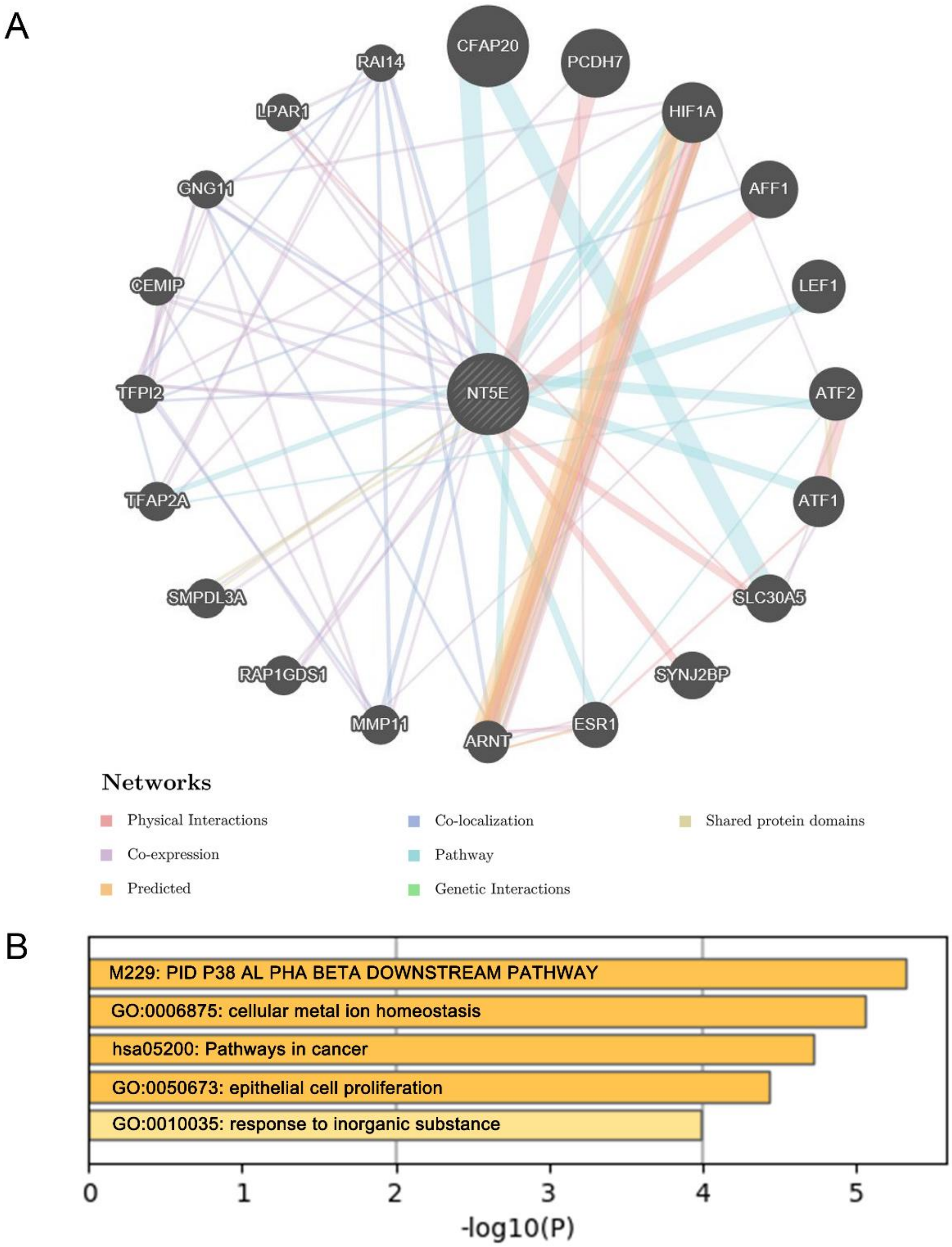

Figure S3. Gene-gene interaction network of CD73/NT5E (GeneMANIA) and functional enrichment analysis. (A) 20 genes related to CD73 using analysis of functional association, including physical interactions, co-expression, predicted association, co-localization, pathway, genetic interactions and shared protein domains, represented by distinct colors of the network edge. (B)

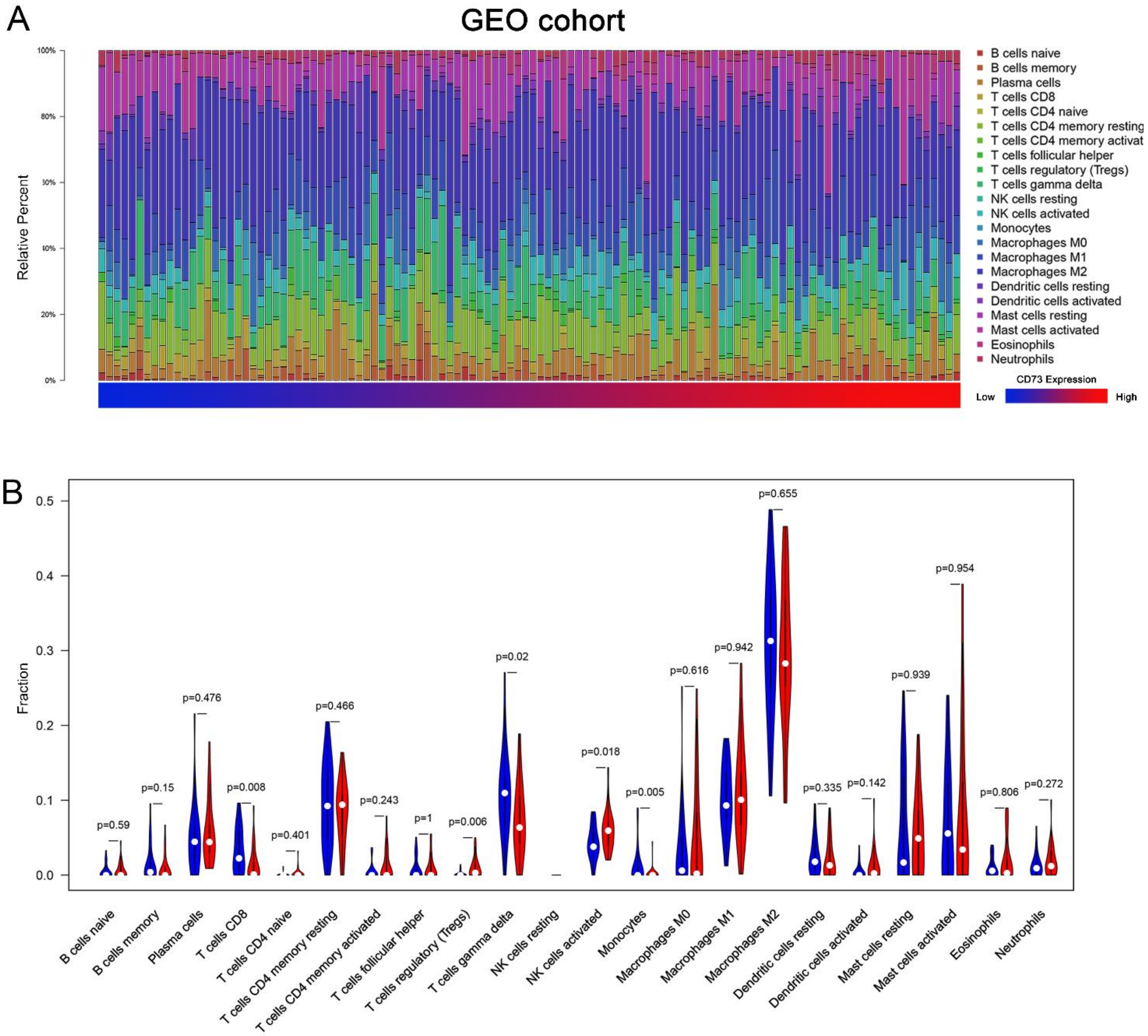

Figure S4. Correlations of CD73 expression with immune infiltration level in the GEO cohort. (A) Fraction of immune cells in 114 tumor tissues arranged by CD73 expression from low to high. (B) Analysis of differential immune cells between the low and high CD73 expression group in GEO.
