# Supplementary material for: CD73 acts as a prognostic biomarker and promotes progression and immune escape in pancreatic cancer
Source: J Cell Mol Med. 2020 Jul 9;24(15):8674–86. doi: 10.1111/jcmm.15500 (PMC7412695; doi:10.1111/jcmm.15500)
Supplement: Supplementary file 2 — Table S1 [file JCMM-24-8674-s002.docx]

**Supplementary Table 1. Correlation between CD73 expression and clinicopathological characteristics of pancreatic cancer.**

| **Variables** | **No. (n=168)** | **CD73 low** | **CD73 high** | **P value** |
| --- | --- | --- | --- | --- |
| **Age**  ≤65  ＞65 | 88  80 | 70  66 | 18  14 | 0.626 |
| **Gender**  female  male | 77  91 | 64  72 | 13  19 | 0.511 |
| **Tumor site**  head  body+tail  unknown | 122  27  19 | 100  23 | 22  4 | 0.906 |
| **Grade**  G1  G2  G3+G4  Gx | 26  91  49  2 | 23  76  35 | 3  15  14 | 0.124 |
| **T classification**  T1+T2  T3+T4 | 28  140 | 25  111 | 3  29 | 0.219 |
| **N classification**  N0  N1 | 50  118 | 45  91 | 5  27 | **0.052** |
| **M classification**  M0  M1 | 164  4 | 132  4 | 32  0 | 0.426 |
| **Stage**  I  II  III+IV | 19  142  7 | 16  113  7 | 3  29  0 | 0.195 |
| **Family history**  No  Yes  Unknown | 43  61  64 | 32  49 | 11  12 | 0.475 |
| **Chronic pancreatitis**  No  Yes  Unknown | 120  13  35 | 94  11 | 26  2 | 0.865 |
| **Diabetes history**  No  Yes  Unknown | 103  35  30 | 80  29 | 23  6 | 0.515 |
| **Alcohol history**  No  Yes  Unknown | 61  95  12 | 53  73 | 8  22 | 0.120 |
| **Neoadjuvant therapy**  No  Yes | 167  1 | 135  1 | 32  0 | 1 |
| **Adjuvant chemotherapy**  No  Yes | 53  115 | 40  96 | 13  19 | 0.219 |
| **Adjuvant radiotherapy**  No  Yes  Unknown | 97  36  35 | 78  31 | 19  5 | 0.448 |
