# Supplementary material for: CD73 acts as a prognostic biomarker and promotes progression and immune escape in pancreatic cancer
Source: J Cell Mol Med. 2020 Jul 9;24(15):8674–86. doi: 10.1111/jcmm.15500 (PMC7412695; doi:10.1111/jcmm.15500)
Supplement: Supplementary file 3 — Table S2 [file JCMM-24-8674-s003.docx]

**Supplementary Table 2. Gene sets enriched in the high CD73 expression phenotype**

| MSigDB colletion | Gene set name | NES | NOM p-val | FDR q-val |
| --- | --- | --- | --- | --- |
| c2.cp.kegg.v.7.0.symbols.gmt | KEGG_PANCREATIC_CANCER | 2.06 | 0 | 0.001 |
|  | KEGG_TIGHT_JUNCTION | 2.03 | 0 | 0.003 |
|  | KEGG_CELL_CYCLE | 2.02 | 0 | 0.002 |
|  | KEGG_ADHERENS_JUNCTION | 2.02 | 0 | 0.002 |
| h.all.v.7.0.symbols.gmt | HALLMARK_MITOTIC_SPINDLE | 2.22 | 0 | 0 |
|  | HALLMARK_G2M_CHECKPOINT | 2.06 | 0 | 0.001 |
|  | HALLMARK_TGF_BETA_SIGNALING | 2 | 0 | 0.002 |
|  | HALLMARK_HYPOXIA | 1.91 | 0 | 0.003 |
|  | HALLMARK_PI3K_AKT_MTOR_SIGNALING | 1.86 | 0.002 | 0.005 |

NES: normalized enrichment score; NOM: nominal; FDR: false discovery rate. Gene sets with NOM P-value <0.01 and FDR q-value <0.01 were considered as significantly enriched.
